# Remarks on the Small-Pox, as It Has Occurred in London Subsequent to Vaccination

**Published:** 1820-07

**Authors:** Rod. Macleod

**Affiliations:** Physician to the Westminster General Dispensary.


THE LONDON
### Medical and Physical Journal.

1 OF VOL. XLIV.] JULY, 1820. [NO. 257.

"For many fortunate discoveries in medicine, and for the detection of numerous "errors, the world is indebted to the rapid circulation of Monthly Journals; "and there never existed any work to which the Faculty in EUROPE and "AMERICA were under deeper obligations than to the Medical and Physical "Journal of London, now forming a long, but an invaluable, series." RUSH.

### Original Communications, Select Observations, etc.

FOR THE LONDON MEDICAL AND PHYSICAL JOURNAL.

*Remarks on the Small-Pox, as it has occurred in London subsequent to Vaccination.* By ROD. MACLEOD, M.D. Physician to the Westminster General Dispensary.

THE records of medicine present nothing at all analogous to the vaccine disease, whether we regard the nature of the thing itself, or the history of its reception. Opposition was indeed attempted; but in such a manner as to render the cause of vaccination more eminently triumphant, by calling forth testimonials in its favour so numerous, and from such high sources, as to command the opinion of the public and obtain the countenance of government. From this moment, all further reasoning may be said to have ended; and the brazen serpent of Moses was not hailed with more enthusiasm, or looked up to with more implicit confidence, than the great discovery of JENNER.

But of late, rumours have gone abroad less favourable to its reputation, and the opinions of many have begun to waver: nor has the epithet "anomalous," bestowed upon the variolоid eruptions occurring subsequent to vaccination, been sufficient to remove their growing distrust. Indeed, at no other period since the discovery of cow-pox has the attention of the community been so anxiously directed to every thing connected with the subject as at present. Under these circumstances, it becomes of importance to enquire coolly into the claims of vaccination, and the grounds on which those claims are founded. And here it is to be regretted, that so much prejudice and party-feeling should be allowed to enter into a discussion which can be determined only by carefully watching the phenomena, and candidly acknowledging our opinions to have been incorrect, as soon as we find them at variance with experience. I am the more inclined to insist upon this, from having observed the solicitude with which some explain away all eruptive diseases, which, though modified by previous vaccination, have yet unquestionably arisen from the contagion of small-pox; while others, deeming the matter long ago decided, are unwilling again to enter into a discussion of its merits, and rather show a disposition to check all investigation by which the accuracy of opinions already admitted is likely to be called in question. In France, indeed, some have gone further, and had recourse to the unworthy artifice of exciting odium against all who presume to doubt this article of medical faith. "Des médecins, à la vérité bien indignes de ce nom, contestent à cette méthode sa vertu préservatrice, et osent employer leurs moyens a attaquer à sapper cette heureuse découverte. Qu'espèrent ils ces amis des vieilles routines? Feront ils retrograder le siècle? Ignorent-ils que les temps ne reculent pas?" Such is the style of argument adopted in a manifesto on the Varioloid Disease, published at Marseilles in the end of 1818, and signed by nine medical practitioners.

As there is no instance of one disease being substituted for another, so it is obvious that, when cow-pox was first proposed as an antidote to small-pox, its pretensions were not founded upon analogy; for all analogy was against it. The writer is quite aware he shall be told that theoretical objections must give place to experience, and that the result of many cases had fully proved the antivariolous power of vaccination before it was admitted, the vaccinated having been found incapable of receiving small-pox even by inoculation. Now this, as far as it goes, is a very curious and interesting fact; but, when it was further argued that such individuals could not have the disease communicated at all, we certainly did not reason with logical good faith: and, accordingly, subsequent observation has refused to acknowledge the legitimacy of the conclusion; many who had resisted small-pox inoculation having taken it by contagion. Besides, general inferences were drawn from cases as yet very limited in point of duration; and, without waiting for experience, it was circulated within less than five years of its original promulgation over Europe, Asia, America, and part of Africa, as an infallible and permanent antidote to small-pox: while it is obvious, that the experience of five years, granting it to have been in every instance favourable, could only prove that, during that period, the vaccine retained its antivariolous power. Whereas, before it could be proved that vaccination was a permanent and general preventive of variola, it would be necessary to have shown that vaccinated persons preserved, in all climates and situations, during the usual period of human life, the power of resisting small-pox, not only from inoculation, but also from contagion; and that, too, not in individual cases, but during the prevalence of epidemics. Epidemics have prevailed; but, unfortunately, the result has disappointed the too sanguine hopes of the friends of cow-pox, though the philanthropist has still ample grounds for consolation in the very mitigated form which the small-pox modified by vaccination almost invariably assumes.

In Dr. Thomson's late work, 1385 cases of varioloid disease subsequent to cow-pox are expressly mentioned as having occurred in Scotland; while several practitioners refer to "many," and some to "a multitude" of cases, without giving the exact number: so that the grand total is probably not under 2000. Besides this, it has prevailed in the counties of Nottingham, Stafford, and Derby, at Liverpool, Norwich, and several other parts of England: in fact, it has become abundantly common both in this country and on the continent.

It is singular, however, that, while our northern and continental neighbours have published various accounts of this unwelcome epidemic, none should hitherto have appeared in London. Those unacquainted with the truth might naturally conclude, from this silence, that here, at least, we had been spared this mortifying proof of the fallacy of our opinions, and give us credit for really possessing some of those superior methods of vaccination to which some among us have not failed to lay claim.* It is now about three years since the number of these cases, which were then held to be accidental, and to proceed from failures in vaccination, first began to excite notice by their increasing frequency; and, during last winter, they became so common, as almost wholly to lose the attraction of novelty. Between the beginning of January and end of May, twenty-three cases of this kind fell under my own observation, and I had previously seen ten; while I have heard of many more from gentlemen connected with public institutions. The disease has likewise occurred at some public schools, where the boys had been previously vaccinated.

The varioloid disease in London appears to have assumed every variety of papular, vesicular, and pustular, eruption, both in form of combination and degree of severity. In general, after an eruptive fever, which is sometimes pretty smart, and for the most part lasts three days, papulæ begin to make their appearance about the face and chest; on the second day, these are more distinct, and feel hard in the skin; and, on the third,

* While the disease was yet rare in the metropolis, it was natural for us to encourage this gratifying explanation; but, unfortunately, too many instances have recently occurred of unequivocal small-pox in persons vaccinated at some of the most reputed establishments in this city, to admit of our indulging it any longer: and, were we so inclined, it is probable that the public voice would soon prove louder than ours.

have generally become vesicular. During the third and fourth days, the fluid contained in the pocks gradually becomes opaque; and on the fifth day crusts begin to form. These are often hard and shining, remaining persistent for several days, sometimes ten, or even more, giving the whole eruption the appearance of little hard papillæ or tubercles. (See Case I.) But, though the great majority of cases fall under this description, yet, in others, the disease assumes every character of distinct small-pox in those who have not been vaccinated, and occasionally becomes coherent, or even confluent. (See Case V.) Yet, even under these circumstances, the eruption frequently retains much of its vesicular character; the pocks appearing, throughout their whole continuance, to be filled with a fluid more serous or lymphatic than purulent; and, where true pustules have been formed, it is not uncommon to see others remain throughout purely vesicular.

The fever which precedes the eruption, is, in many instances, as severe as that which ushers in inoculated or mild natural small-pox : sometimes, indeed, it runs high, with much anxiety and delirium. In the case of a young gentleman, whom I lately saw with my friend Dr. Ashburner, the eruptive fever assumed the typhoid type, and was accompanied with numerous petechiæ on the neck and chest; a circumstance which, he informed me, he had seen in another case, where the eruption proved confluent. In one instance, I have seen several vesicles filled with a purple fluid, and a considerable number of the pustules surrounded with a little purple areola, as if they rose from the centre of petechiæ. (See Case V.)

On the appearance of papulæ or vesicles, the constitutional derangement generally subsides or disappears, and the pocks often come out for three or four successive days; but few of them advance to the state of vesicles or pustules, at least when compared with the original eruption. Sometimes these crops appear on the eighth or ninth day ; and, in the young gentleman above alluded to, they occurred as late as the fourteenth. The pocks vary much in size, but, upon the whole, they are decidedly smaller than in other forms of small-pox, seldom exceeding half a split-pea in diameter: they are, for the most part, larger when purely vesicular, than when they have much of the papular character. They frequently want the central depression ; but the occurrence of this appearance seems unconnected with the size of the pocks, for I have observed it all over the body in pustules which never became larger than pins' heads, and have seen a thick crop of pustules all over the body larger than is usual in the distinct natural disease, without any depression. These remarks, which are drawn from cases occurring in London, will be found to agree in almost every particular with the epidemic in Edinburgh. A phenomenon, however, is described by Dr. Thomson, which, as far as I know, has rarely been observed in this town: I mean the recurrence of the disease; and one instance is mentioned in which this happened twice. "In above forty of those who had been previously vaccinated, the varioloid disease has occurred for the second time, at intervals, varying from a few days to several years. In some of these cases it exhibited, in the first attack, the appearance of chicken-pox, and in the second that of small-pox: in others again, in the first attack it resembled small-pox, and in the second chicken-pox. In some the disease has in both attacks resembled chicken-pox, and in others small-pox. I have seen but one instance of a person who had been vaccinated having the varioloid disease for a third time."*

Several causes have been assigned as likely to have acted in producing many of those cases of what are termed failure of the cow-pox: such as imperfect vaccination, deterioration of the lymph, and, lastly, the antivariolous effect becoming impaired or destroyed by time. Much stress is laid upon the first of these in some of the late reports of the National Vaccine Establishment, in allusion chiefly, it is supposed, to the system of testing the vaccine vesicle, as recommended by Mr. Bryce. That gentleman's plan consists, it may be remembered, in making one puncture, and on the fifth or sixth day inoculating the other arm from the vesicle thus excited. If the constitutional effect has been produced, it is found that the second inoculation gives rise to a vesicle which anticipates the usual course, and, if I may use the expression, overtakes the first. Mr. Moore, on the other hand, advises, as a general rule, to make two punctures in each arm: if all these punctures succeed, then we are told that lymph may be taken from the patient; "but it is always prudent to leave two complete vesicles to run their course unmolested." This plan of saturating the system with lymph is like reducing vaccination to a chemical process, though it is not easy to conjecture how it was discovered that the capacity in the human constitution for variola was too great to be neutralized by one vesicle, and yet became supervaccinated by two. The nugatory nature of the evidence upon which the claims of this plan to such high superiority are founded, is so well illustrated in the able review of Dr. Thomson's book in the Edinburgh Medical and Surgical Journal for April, that it is unnecessary to enter into the discussion here. I

* Since these remarks were begun, a child whom I attended in January for what I then regarded as small-pox subsequent to vaccination, has again become my patient for a varioloid eruption, which has successively been papular, vesicular, and pustular. In this case, to which I shall have occasion to allude again, the child was vaccinated four years and a half ago, at the Institution in Lisle-street, and has five perfect cicatrices upon his arms.

may however be permitted to remark, that I have seen too many instances of small-pox in children vaccinated in London, where that process was carried on in the way which the National Vaccine Establishment has recommended as most efficacious, to retain much faith in its preventive powers, in whatever manner conducted; for the circumstance just mentioned, renders the plea of imperfect vaccination inadmissible in the cases to which I allude.

The second conjecture, viz. that the vaccine lymph may have become deteriorated in passing through so many human constitutions, is one in the face of all analogy, and, as far as it is possible to judge at present, seems wholly unfounded; at least, if identity in external character is to be regarded as indicating similarity in its occult effects. I am aware, however, that some are disposed to think an alteration in the size and appearance of the vesicles is perceptible; but the result of some recent observations has led me to believe that the size of the cow-pock may be modified to a certain extent by the nature of the puncture; and, on examining a considerable number of children in different stages of vaccination, I have found the size of the vesicles vary perceptibly on the same arm, though excited by inoculation with the same lymph and instrument. So that, in truth, it seems more likely that the diminution in the size of the vesicles is rather to be attributed to the extremely minute puncture by which some insert the matter.

The vaccine lymph has not been changed at the Small-pox Hospital since the year 1799. During this period of more than twenty years, the number vaccinated up to May 19, 1820, amounts to 47,726; yet I am informed by Dr. Ashburner, that the late Mr. Waschell, who was apothecary to the hospital at the time cow-pox was first introduced, so far from seeing any difference in the vesicle, or deterioration of the lymph, regarded it as improved by this process, and took especial care to preserve the original chain from being broken. Dr. Thomson likewise informs us, that the vaccine virus which has been used in the Dispensary in Edinburgh for eighteen years, still produces the very same appearances in those inoculated with it as it did on its first introduction; and further, that this virus, which he calculates to have passed through a succession of at least nine hundred individuals, produces vesicles exactly similar to those excited by recent vaccine matter sent to him by Dr. Jenner. Now, if we were to allow that the lymph, in passing through only nine hundred systems, had become deteriorated to such a degree as to afford any explanation of the late frequency of modified small-pox in Edinburgh, it is quite obvious that the lymph at the Small-pox Hospital, having passed through so much greater a number, must have degenerated so as to leave by this time very little antivariolous property: whereas, there is no evidence, nor any reason whatever, to suppose it inferior to any other in London.

The third imagined source of small-pox after vaccination, is, that cow-pox by time loses its antivariolous properties; and, as this view is calculated to excite anxiety in all who have trusted to the permanency of its operation, so there is no point on which the medical man is more frequently questioned; an idea being afloat, that, in order to be quite secure, it is necessary to undergo the process every seven years, or at some other period expressly specified. I believe there is no analogy by which this cessation of vaccine influence can be supported, and general observation seems against it. I therefore must protest against the accuracy of a contemporary writer, who says, "We are sure that, generally speaking, it is not a common occurrence before the tenth year; and the average of our cases have happened twelve or thirteen years after vaccination." Now, as we are not favoured with the number of cases from which this average is drawn, it is impossible to determine how far it is entitled to be admitted as a standard; but, from all that has hitherto been published on this subject, I suspect that, generally speaking, it is more common to see the interval between vaccination and small-pox under ten years than above it. In support of which position, I subjoin a table showing the intervals in 251 cases, from the work of Dr. Thomson.

| Interval. | No. | Interval. | No. | Interval. | No. | Total. |
|---|---|---|---|---|---|---|
| 1 Month | | 9 | 7 | 9 | 19 | |
| afterVac. | 2 | 1 year | 26 | 10 | 15 | |
| 2 | 3 | 2 | 16 | 11 | 15 | |
| 3 | 1 | 3 | 13 | 12 | 15 | |
| 4 | 2 | 4 | 13 | 13 | 7 | |
| 5 | 1 | 5 | 15 | 14 | 12 | |
| 6 | 7 | 6 | 15 | 15 | 7 | |
| 7 | 2 | 7 | 16 | 16 | 2 | |
| 8 | 1 | 8 | 17 | 17 | 2 | |
| | 19 | | 138 | | 94 | 251 |

From this it appears, that 79 only had the disease at so great an interval as ten years after the cow-pox, while 109 had it within five years; and the average of my own limited observations would make the interval still shorter.

Whether the cases which appear to decrease in frequency after the first year, as we recede from the period of vaccination, may not increase, as some have supposed, in point of severity, is quite another question. The longest interval I have seen is fourteen years; and the disease, which in this instance occurred in a young woman in the ninth month of pregnancy, was very much modified, and such as, under other circumstances, would have been regarded as genuine varicella. It arose from having slept with a child labouring under natural small-pox; and her infant, when born, was not in any way affected by it. (See Case IV.) On the other hand, in one of the most severe varioloid eruptions subsequent to vaccination which has fallen under my observation, and to which I have already alluded as ushered in by high fever and delirium, the interval was only two years. (See Case II.) Dr. Thomson, whose opinions upon this subject must be regarded as entitled to high consideration, informs us, that he has seen nothing which would induce him to suppose that the antivariolous powers of vaccination are affected by time: so far from it increasing, years appear to him to diminish the susceptibility to the contagion of small-pox. (p. 34.)

From speaking of the comparative severity under different circumstances, we naturally pass to another very interesting question, viz. the rate of mortality in cases of small-pox after vaccination. This, however, is a point by no means easily ascertained. In Edinburgh, only one died out of 330: but, if we reverse the proposition, and suppose that, for every fatal case in London, 329 have had it in a milder form, I believe we would make the actual number greatly above the truth. Four of Dr. Thomson's correspondents mention having seen one fatal case each; several are well known to have occurred in this town; and the National Board acknowledge five deaths having been reported to them, but are inclined to suppose they originated in imperfect vaccination. A minute account of a case of fatal varioloid disease subsequent to vaccination, I have reason to believe, will appear in the same Number of the Journal which contains this paper; and, among the cases illustrative of different points connected with this subject, will be found one, (No. VI.) which terminated unfavourably; but, as the circumstances were peculiar, I am not inclined to add it to the list of deaths from small-pox. Dr. Gaspare Ghirlanda, in an account of the varioloid epidemic which prevailed at Treviso in 1818, states, "Che il vajuolo, che si fa vedere in qualche vaccinato è più benigno di quello che si svolge dall'innesto umano, e quasi affatto scevro di periculo, imperciocchè in tutto il mondo appena si possono raccogliere sei casi di morte, mentre dall' innesto umano ne muore uno in quattrocento." Certainly the mortality from modified small-pox is extremely small; but it will be fortunate, if more extended observation does not lead Dr. Gaspare Ghirlanda to suspect that he has rated it too low.

The history of vaccination altogether forms a severe satire upon the mutability of medical doctrines. In the first ardour of discovery, not contented with its blessings to mankind, its benefits were also extended to the brute creation: it was to annihilate small-pox,* prove an antidote to the plague, to cure the rot in sheep, and preserve dogs from the mange. These good-natured speculations, however, were soon abandoned; and, more recently, all had agreed in acknowledging its anti-variolous powers, which, we were told, were as well established "as any thing human could be." But the present epidemic shows too clearly the mortifying fallibility of medical opinions, though founded on the experience of twenty years, and guaranteed by the concurring testimony of all the first physicians and surgeons in the world. I beg not to be misunderstood. So far from entertaining an opinion unfavourable to vaccination, I am inclined to think the comparison of small-pox in its modified and unmodified forms, which has recently been forced upon the notice of the public, will be sufficient to satisfy all reasonable people of its great utility. But, then, it seems mistaken policy in medical men to persist in attributing to cow-pox the power of preventing a disease which too frequently occurs after it; because persons ill-informed, or prejudiced, finding their assertions partly incorrect, are naturally led to reject them altogether.

Before entering upon the second part of this paper, in which it is my intention to offer some remarks upon the common origin of the various forms of varioloid eruptions, I shall relate a few of the cases which I have regarded as small-pox subsequent to vaccination; and, should any one be inclined to object to the accuracy of this opinion, I beg it may be observed, that, of the two mildest, Nos. III. and IV. one arose unequivocally from the contagion of small-pox; and the other, by inoculation, communicated that disease. It may be proper farther to mention, that the cases from which these observations have been made, were seen, with the exception of No. VI. by one or other of the following gentlemen: Dr. Harrison, Dr. Ashburner, Dr. Gregory, Dr. Hutchinson, some of my colleagues, and the pupils at the Westminster General Dispensary.†

---

* That the introduction of vaccination should have given rise to plans for extirpating the small-pox, is by no means astonishing; but, what shall we say of the intrepidity of a German physician, who undertook this before he had heard of cow-pox, and in the face of eighty-four objections! "In hoc opere viam designasse mihi videor, qua quidem medicis illam incedentibus certissime datum foret, sucessivam preparare et ad finem perducere variolarum exterpationem, et *octuagenta quatuor* objectiones huic exterpationi oppositas refutavi, ad viam nempe dictam respiciens."—See a letter from Dr. J. C. W. Juncher to Dr. Pearson, in the fourth volume of this Journal.

† The great extent of space occupied by the articles we have been obliged to appropriate for insertion in the present Number, renders it necessary that we should defer that part of the paper of Dr. Macleod above alluded to, until the

### Cases *illustrating some of the Appearances assumed by Small-Pox after Vaccination.*

Case I.—Thomas Lucas, aged five years, residing at No. 25, New Compton-street; vaccinated, when six months old, in Lisle-street.

*May 16th.*—Has been affected with smart fever for three days. This morning a papular eruption made its appearance on the face, neck, chest, arms, and legs, which promises to be crowded. One on the left fore-arm has passed into the form of vesicle with central depression. Fever still considerable.

17.—Papulæ numerous and well-formed on the body and limbs, feeling hard in the skin when the finger is run over them. Fever continues.

18.—Papulæ for the most part crowned with little vesicles, with some appearance of central depression. Fever abating.

19.—Eruption becoming pustular. Some of the papulæ seem disposed to go back. Fever gone.

20.—Some of the pustules about the face becoming covered with crusts, a few of the papulæ assuming the appearance of little horny scabs.

21.—Pocks all over the body covered with crusts of a hard shining appearance.

23.—Some of the crusts dropt off, leaving blains.

27.—Little smooth reddish tubercles are the only remains of the eruption.

Case II.—Selina Dove, aged nine years, residing at No. 7, New Round-court, Strand. Became affected, February 18, 1818, with fever, great anxiety, head-ach, and delirium; which symptoms were treated as an attack of the epidemic fever which prevailed at that time. On the third day a copious crop of papulæ was observed on the face, chest, and extremities, but particularly crowded on the face. On the appearance of the eruption the fever subsided, and did not again return. The papulæ gradually enlarged, and in two days were crowned with vesicles, which by the fifth had passed into the form of perfect pustules, without any central depression. From this time the eruption began to pass away in the form of crusts, which, dropping off in a few days, left blains, but no pitting. While she was convalescent, her brother, aged six, passed through a varioloid disease, similar in nature, but milder in its form.

Both these children had been vaccinated two years prior to this attack, at the Jennerian Institution in Holborn.

Case III.—Henry Oldfield, aged seven years, residing at No. 2, Rose-street, Soho. Vaccinated at the Small-pox Hospital when a few months old, and having a perfect cicatrix on the arm.

*March* 21.—Was seized on the evening of the 19th with head-ach

---

ensuing month; and, as it relates to so distinct a view of the diseases under consideration, we think it may, without impropriety, notwithstanding its interesting nature, be separated from that which is here introduced to our readers.—Edit.

and considerable fever, which still continues. Small papular points are making their appearance about the face, chest, and back.

22.—Papulæ almost all converted into little vesicles filled with straw-coloured lymph. Fever much abated.

24.—Contents of the vesicles becoming opaque.

*April* 1.—Crusts began to form on the 25th: such of the papulæ as had not become vesicular went back, and the whole eruption has now disappeared.

Two children residing in the same house were inoculated on the 24th, from this eruption, in consequence of their being unavoidably exposed to its contagion, and the absolute refusal of their mother to allow them to be vaccinated. Both took the usual form of inoculated small-pox.

Case IV.—Lucy Stillwell, aged 22, in the ninth month of pregnancy, vaccinated fourteen years ago at Oatley, and having a perfect cicatrix on the arm, residing at No. 4, Russell-place, Bow-street; eight days after sleeping with a child labouring under natural small-pox, the smell of which was loathsome, and made a strong impression upon her, became affected, February 5th, with nausea, head-ach, and considerable fever, on the third day. Little red papular points were observed, and the fever began to decline, but did not quite leave her for two days. On the second day the eruption had become vesicular, with little, if any, surrounding induration, but with a considerable blush of inflammation. The whole number of pocks was inconsiderable, consisting of one upon the hand, five or six upon the breast, and a cluster of eight or ten upon the nates. During the third and fourth days, the vesicles became gradually more opaque; on the fifth, crusts began to form; and from that period the eruption rapidly disappeared.

Her infant, born March the 3d, was not in any way affected.

Case V.—William Pyrhe, aged eight years, residing at Clerkenwell, vaccinated at the Small-pox Hospital when a few months old, and having a perfect cicatrix on his arm, was seized on Thursday, 18th May, with violent fever, intolerance of light, head-ach, and delirium, accompanied with extreme restlessness and fits of screaming. His medical attendant, apprehending inflammatory mischief in the head, applied leeches to the temples and a blister to the back of the neck, by which some relief was afforded. On the 19th, some red points were observed on different parts of his body, which by the next day had passed into a papular eruption on the face, neck, chest, abdomen down to the umbilicus, arms, and legs; the back and abdomen, from the umbilicus to the pubis, being free from eruption. The constitutional derangement abated on the 20th; and on the 22d the papulæ had passed into the form of opaque vesicles. To-day (24th), the eruption presents the appearance of a thick crop of pustules all over the body, with the exception already mentioned; on the face, they are confluent round the mouth and under the eyes, and coherent in other parts. On the fore-arms many of the pocks are surrounded with a narrow purple areola, giving the appearance of pustules rising from the centre of petechiæ. Several of the pocks are filled with a purple fluid, and there is a large one of this kind upon the left cheek. The pustules are in general small, some not exceeding a pin's head, and the largest scarcely the size of a split-pea. The boy cannot yet stand.

26.—Crusts have formed in the site of the pustules all over the body, and the boy is getting rapidly well.

There seems to be what might be called a variolous diathesis in this family, as another of the children had a varioloid eruption, in a milder form, subsequent to vaccination, and the father had small-pox twice; the first attack having left him pitted all over, and the second, which he caught from a sister then labouring under the disease, having also left several marks.

Case VI.—Robert Page, aged one year and nine months, residing at No. 6, Tower-street, was affected with fever and general indisposition for several days prior to the 21st January, when a rubeoleous eruption made its appearance, and continued for three days; disappearing again on the 23d. The child continued restless, with some diarrhœa and much fever. Next day (24th), an eruption of papulæ was observed upon the back, which in a few hours became vesicular, being filled with a fluid of a bluish colour, so as to give them a pearly appearance. They had a central depression, and, except becoming rather more opaque, maintained the same character throughout. The constitutional symptoms did not abate on the appearance of the eruption, and the child was affected with occasional convulsions. These became more severe on the 28th, when a fresh vesicle, similar to those already described, came out on the thumb; and in the evening the child died.

—— Page, aged three, was affected, at the same time as his brother, with a rubeoleous rash, which ran the usual course of measles. On the 26th, two papulæ came out on the right fore-arm, which quickly became vesicular, with central depression. In a few days they became covered with crusts, which remained persistent for fourteen days, and, on dropping off, left pits.

Both these children had been vaccinated at Broad-street, and were supposed to have had the disease in a satisfactory manner. Small-pox, in its natural form, prevailed in the adjoining house. What was the nature of the second eruption in these two children?

*Great Marlborough-street; May 27th*, 1820.

---

FOR THE LONDON MEDICAL AND PHYSICAL JOURNAL.

